# An Epitome of the History of Pathology*Introductory Lecture of the Course in Pathology at the Atlanta Medical College, 1896-97.

**Published:** 1896-11

**Authors:** Charles Minor Blackford

**Affiliations:** Atlanta, Ga.


### Southern Medical Record.

A MONTHLY JOURNAL OF MEDICINE AND SURGERY.

VOL. XXVI. ATLANTA, GA., NOVEMBER, 1896. No. 11.

### Original Articles.

### AN EPITOME OF THE HISTORY OF PATHOLOGY.*

By CHARLES MINOR BLACKFORD, JR., M. D., ATLANTA, GA.

GENTLEMEN:

It is my object, before beginning the regular work of the session now opening, to give you a short sketch of the rise of our science from the faint glimmer of light found at the very dawn of history to the present time, that you may see the difficulties that beset our professional ancestors, the way in which they were overcome, and the gradual conquest of ignorance, superstition and bigotry. In this manner it is my aim to inspire you with an admiration for those pioneers in intellectual advancement, those martyrs who often paid with their blood for their temerity in breaking the narrow limits to which an unappreciative world would have bound them, and who were fortunate if merely ridicule and abuse became their lot. With but few exceptions, the lives of those to whom we are most indebted were passed in poverty, neglect or persecution, and their rich harvest of posthumous fame is an inadequate reward for the great blessings bestowed on mankind by their toil.

*Introductory Lecture of the Course in Pathology at the Atlanta Medical College, 1896-97.

Pathology, the science of disease, its causes and effects, has been the greatest sufferer from superstition and ignorance. The respect shown to the human body, even after death, has been an obstacle to its study not yet fully overcome. Human skill seemed so powerless in the presence of great plagues and pestilences that it was not unnatural that they were attributed to supernatural agencies which it was worse than useless to investigate.

The nation of antiquity of which we have most full and accurate accounts is Egypt. Not merely were the Egyptians a learned people, but owing to many different reasons their records have descended to us in greater perfection than have those of any of their cotemporaries, and owing to the great natural wealth of the country and the consequent trade relations with the other parts of the world, there are few writers of antiquity who do not mention this strange country. Our knowledge of their system of medicine is more complete than that of any of the ancient empires, and an outline of it forms a proper introduction to our subject.

The learning of the Egyptians was recognized as pre-eminent by all of the peoples with whom they came into contact. In Homer we read:*

> "These drugs, so friendly to the joys of life,
> Bright Helen learn'd from Thone's imperial wife,
> Who sway'd the sceptre where prolific Nile
> With various simples clothes the fatten'd soil.
> * * * * * * * * * *
> From Pæon sprung, their patron god imparts
> To all the Pharian race his healing arts."

In Jeremiah we read: "Go up into Gilead and take balm, O virgin daughter of Egypt; in vain dost thou use many medicines; there is no healing for thee,"(Jer. 46:11,) and Kings Cyrus and Darius sent to Egypt for physicians.† Heroditus gives many interesting accounts of these physicians. They were paid stipends from the public treasury, and were expected to treat the poor and the soldiers of the army gratuitously in consequence, but were allowed to collect fees from their wealthy patrons. They were required to treat their patients

*Pope's Translation of the Odyssey, Book IV.

†Heroditus. History translated by Rawlinson; Book II., chapter 84, and notes.

in accordance with the precepts laid down in the books, or be liable to capital punishment in case of the death of the sick one. As new remedies forced themselves into use, they were slowly adopted, and the chief use of the old rule was to prevent experimentation on the part of young physicians. Aristotle* says that after the third day without improvement, the physician was allowed to change the treatment, or even earlier at his own risk. Dentistry was practiced with no little skill, and mummies are found with the teeth filled with gold, although we generally consider that operation a modern one. There seems not to have been a sharp distinction between embalmers and physicians, for Joseph called the physicians to embalm his father Jacob.† They were recognized as belonging to the priesthood and of an order called Pastophori, and were expected to know all things relating to the body, diseases and remedies, as laid down in the last six sacred books of Hermes. The profession was far from an ignoble one, members of the nobility, and even royalty itself, being from time to time enrolled in it. Athothes, the second king of the first dynasty, was a physician and wrote works on anatomy, and his name, corrupted into Hermogenes by the influence of the Greek, gave rise to the idea that they were written by Hermes, the Egyptian Thoth.‡

Diseases were treated by drugs, aided by charms and incantations.§ Magical words were written on strips of papyrus and carried by the patients, or the magic formulæ were chanted by the healers. Dreams were considered as foretelling the result of the treatment, and the soothsayers had an elaborate system of interpretation for them. Ateta, the third king of the first dynasty, wrote a work on anatomy, and discovered an infallible wash to prevent the hair from falling out, which, with commendable filial piety, he dedicated to his mother.

Medical works abounded in Egypt from the earliest time, and some five hundred manuscripts bearing on medical subjects are now known to exist. Of these the most valuable is the "Ebers papyrus," brought to Europe by Dr. Ebers in 1874,

---

*Aristotle. Polit. III., 11.

†Genesis, 50:2.

‡Rawlinson, note on Heroditus cited above.

§Ebers. Egypt, Vol. II., pp. 61-62.

which is an encyclopedia of medical lore. It contains one hundred and ten pages, each bearing twenty-two lines of bold hieroglyphic writing, and is made up of receipts for the cure of all diseases, some borrowed from the Syriac, and some of such antiquity that they date from the time when the gods moved on earth with men. The remedies were horrible in the extreme. Raw meat figures in several, and a sovereign specific was composed of nitre, beer, milk and blood boiled together and taken hot. The bones, fat and skin of vultures, bats, lizards and crocodiles had varied virtues, as also had the bile of certain fishes.* With such remedies, it is not surprising that magic was preferred.

Such being the treatment, it will be interesting to see the pathology on which it was based. Says Maspero: "Man does not die, but some one or something assassinates him. The murderer often belongs to this world and may be pointed out. * * * Often it belongs to the invisible world, and only reveals itself by the malignity of its attacks: it is a god, a spirit, the soul of a dead man that has cunningly entered a living person or that throws itself upon him with irresistible violence. * * * Whoever treats a sick person has, therefore, two equally important duties to perform. He must discover the nature of the spirit in possession and, if necessary, its name, and then drive it out or even destroy it. He can only succeed by powerful magic, so he must be an expert in reciting incantations and skilful in making amulets. He must then use medicine to contend with the disorders which the presence of the strange being has produced in the body; this is done by a finely graduated regime and various remedies. The cure-workers are, therefore, divided into several categories. Some incline toward sorcery, and have faith in formulæ and talismans only; they think they have done enough if they have driven out the spirit. Others extol the use of drugs; they study the qualities of plants and minerals, describe the diseases to which each of the substances supplied by nature is suitable, and settle the exact time when they must be procured and applied; certain herbs have no power unless they are gathered during the night at the full moon, others are efficacious in summer only; another acts equally well in winter or summer. The

*A. B. Edwards. Pharaohs, Fellahs and Explorers, Chapter 6.

best doctors carefully avoid binding themselves exclusively to either method."*

From this brief notice it may be seen that the Egyptian physicians possessed a considerable amount of skill in surgery and a respectable knowledge of anatomy, as might be expected from their custom of embalming the dead. They probably invented and certainly used the moxa and other counter-irritants, and to their ingenuity the use of artificial limbs is also attributed. Operations on the eye were performed, and the instruments used for the purpose are shapely and well-made. They held some rational ideas in regard to the causes and nature of diseases, but these were so blended and mingled with the grossest superstitions, and bound by such rigid conservatism, that true advance was impossible.

The great fame of the Egyptian physicians acted as a deterrent on the nations with which they came into contact, and in Assyria and Babylon there seem to have been no regular physicians at all. When an Egyptian could not be had, the patient was borne to the market-place and laid on a cot in view of all the people, who then crowded around and offered their advice, based on the previous personal experience of the adviser. Anyone who has been so unfortunate as to have a "cold in the head" can appreciate the feelings of such a sufferer at the close of this treatment. The astrologers ruled supreme in the land and there was no room for another science, and if the "*vox populi*" proved not to be the "*vox Dei*," a magician was summoned. Some of the incantations have been preserved on the clay tablets, and give us curious examples of the thought of the time. Space does not permit the introduction of many of them, but one, intended to cure "disease of the head," which appears to have been erysipelas, is worth repeating. It is as follows:

"The disease of the head exists on this man.
The disease of the head, the ulceration of the forehead, exists on this man.
The disease of the head marks like a tiara, from sunrise to sunset.
In the sea and the vast earth a small tiara is become the tiara, the large tiara, his tiara.
The disease of the head pierces like a bull,
It shoots like the palpitation of the heart.

*Maspero. Life in Ancient Egypt and Assyria, Chapter 7.

The disease of the head, like doves to their dovecotes,
Like grasshoppers into the sky, like birds into space,
So may it fly away. May the sick man be replaced
In the protecting hands of his god."*

Among the Hindoos we find a mixture of knowledge and ignorance, of liberality of thought blended with the most slavish obedience to superstition, even more striking than among the Egyptians. Charms and spells had less weight and the natural forces were more studied than on the banks of the Nile, but the occult forces were still invoked and "the powers of the air" revered and feared. The materia media of the Hindoos was voluminous, and the symptoms produced by the varying doses of the many drugs with which they were acquainted, were described carefully and with painfully accurate minuteness. In surgery their skill was very great but confined to narrow limits. The operation of rhinoplasty has been adopted into our practice from these Indian surgeons, but with this exception we have learned but little from them.

Great attention was paid to diet, and a special name, Pathyapathya, was applied to this branch. The medical works are collected in the Ayur Veda, a work of great antiquity and authority, which is considered as forming a part of the fourth or Atharva Veda, and as being the work of Brahma himself.† Among all semi-barbarous people the surgeon was of greater utility than the physician. In Homer we notice that Podalirius and Machaon were not consulted during the prevalence of the plague, though they were regularly employed to treat the wounds made by darts. The skill of the Hindoos in surgery declined for some reason, and at present is not practised at all.

The chief importance of Hindoo medicine to us is derived from the influence exerted by the race on the Arabs, to whom we are indebted for the rise of modern science; but as many of the ideas of the Arabic sages came indirectly from the Greek, as will be shown in this lecture, a further discussion of this topic will be deferred.

The Greeks were the most cultured and intellectual of all the nations of the olden time, and among them the physical sciences attained prominence as subjects of study. Hippocrates,

*F. Lenormant. Chaldean Magic, Chapters 1 and 3.
†H. H. Wilson. Essays on Sanscrit Literature, pp. 269-276.

Galen and others exerted an influence on medical thought that has persisted almost to the present day, but, unfortunately, with much that was valuable were entwined many errors which were difficult to overthrow because of the high standing of the authors responsible for them. The metaphysical tendencies of this imaginative, poetical and artistic people gave rise to many remarkable theories of nature, traces of which still survive. The notion that the soul resided in the air that was inspired and expired, and that it permeated the body through the arteries, has left its mark in the name of those vessels, and from the fact that the left side of the heart was usually empty after death arose the popular idea, still preserved in poetry, that the heart was the seat of the affections and most of the intellectual faculties. Aristotle maintained that the function of the brain was to cool the blood, but it was well in comparatively modern times that even this approach to physiology was made. The healing arts were from earliest days considered as falling in the province of the priests, and the rise of them was ascribed to the gods. The mythical Æsculapius was usually accounted the founder of medicine, and his two sons, Podalirius and Machaon, figure as the surgeons of the Grecian forces before Troy. Probably in the manner mentioned above the god Hermes was credited with writing some of the sacred books, but in whatever way the idea arose, it was to the temples and the priests that the people resorted for healing. Most of these temples were erected in the neighborhood of *thermæ*, or warm springs, and the treatment consisted chiefly in bathing in and drinking the waters of the sacred fountains, accompanied by prayers to the gods, and muttered charms. Systematic rubbing similar to our massage, either with the bare hands of the priests or with decoctions of odoriferous drugs, rigid diet and careful exercise, made up the course, and the results were often excellent, especially in gouty or rheumatic troubles.

As Athens attained its greatest grandeur and wealth, culture and education became spread among the citizens, the belief in the old mythology gradually passed away, and the sacred element in medicine passed away with it. A new school of writers arose who had materialistic views, who looked on the human body as a piece of mechanism, and who advocated materialistic means of curing its diseases. Chief of these was Hippocrates.

Born on the island of Cos about 460 B. C., of the line of the Æsculapiads, the alleged descendants of Æsculapius, he was early designed for the ancestral profession of medicine. With this in view, he was sent at a tender age to Athens, where he had for cotemporaries Plato, Socrates, Heroditus, Thucidides, and Gorgias. Amid the intellectual life of Athens, at its most brilliant period, he was taught logic, physics, geometry, astronomy and philosophy, as well as the medical science of the day, and in these schools he lingered many years. On his return to his native island, his father, Hericlides, taught him the traditional lore handed down from generation to generation in his family, and the young physician commenced practice in his home. For several years he remained under the direction of his father, but on the death of the latter he determined to travel to enlarge his store of information. For twelve years he wandered through Greece and Asia Minor, learning the properties of all medicinal substances, making experiments and profiting by those of others. He by no means confined his attention to medicine, but studied every science then known. His works cover a multitude of topics, and apparently touch each but lightly, though an attentive reading shows that on each he is as profound as the learning of his day permitted. "The perusal of the works of Hippocrates is, to this day, one of the most instructive exercises to which we can apply, not that the facts that are found contained in them have not been revised by the moderns in collections that are infinitely more ample and perfect, but because no other writer, without exception, initiates us so far into the mysteries of nature, or teaches us to interrogate her with that wise caution, and that scrupulous attention, which alone can enable us to trace from her answers those principles and rules which must always be recognized as genuine."*

Aside from his literary work, he stands before us as a skilful, bold, and successful surgeon. He often opened the skull with the trephine, and did not hesitate to cut into the chest to evacuate collections of fluid. His views on pathology, although now seen to have been erroneous, were superior to those held

*Pettigrew. Memoirs of Physicians, Surgeons, etc.

previously, as any clearly-stated view is preferable to an ill-defined one. He maintained the existence of certain "humors" in the body which were responsible for disease. When illness occurred, the humors became thick before they could be eliminated from the body, and this thickening he called a coction. The effort made by the system to effect a coction, that is, a combination of the morbific matters in the body, gave rise to disease, and the climax of this process followed by the expulsion of the offending material, was called the crisis.* This was the first formulation of what we may call a system of pathology, and appeared to be borne out by clinical observation, and made so deep an impression on medical thought that traces of it may yet be discovered.

The constant intercourse of the Greeks with the Egyptians had an effect on Greek practice, but the mental characteristics of the two races kept them at a distance. The Egyptians ascribed everything to the influence of their gods, and their medicine was blended with superstition throughout, whereas the Greeks had but little faith in gods, and their polytheism offered opportunities of adding to their deities whenever convenient. Their philosophic tendencies led them to study nature and to recognize nothing as supernatural, though they appreciated their own ignorance of the phenomena of nature itself. They were ready to learn from any source, and their medical practice became a blend of everything that experience showed to be good from all the nations about them.

It is not my intention to attempt a full account of Greek medicine, but some few of their cures will be of interest. The herb alysson was supposed to have marvelous virtues. Pounded up and eaten with meat it would cure hydrophobia. A piece of it suspended in a house would protect the inmates from disease and witchcraft, and wrapped in a bit of scarlet flannel and suspended around the neck, it preserved the wearer from all diseases. Saffron, made into an ointment and rubbed on the nostrils, was a remedy for insanity, as was also hellebore. This opinion survived almost to our time, as it is mentioned by Shakespeare in this connection. A roasted scorpion was curative of its own bite, as was also a powdered sea-crab. Vipers contributed their share to the materia medica, for,

*Roswell Park. Lectures on the History of Medicine. (In manuscript.)

caught alive, they were put in a crucible with salt and powdered figs and heated in a fire until the contents of the crucible were reduced to charcoal. This had rare medicinal virtue, and until the last century viper's flesh was a distinct article of commerce, being brought from Egypt to Venice for use in a medicinal ointment. The ashes of old leather cured burns, corns, and blistered feet, and nitre, dissolved in beer, was used in many conditions accompanied by fever. Their surgery was good. As viewed in the light of our times, we see that antiseptics were constantly applied, though the surgeon had no idea of the true principle on which the balsams and distillates that he applied really had their healing influence.

Among the Hebrews the ounce of prevention was in far more use than the pound of cure. The sanitary code, as laid down in the books of Moses, is unequaled in the world, and so long as it was observed the physical welfare of the Jewish people was excellent. Viewed in the light of modern bacteriology, the directions given for the avoidance of infectious diseases are wonderfully adapted to the purpose, as a single example will show:

"When ye be come into the land of Canaan, which I give you for a possession, and I put the plague of leprosy in a house of the land of your possession; then he that owneth the house shall come and tell the priest, saying: There seemeth to me as it were a plague in the house; and the priest shall command that they empty the house, before the priest go in to see the plague, that all that is in the house be not made unclean; and afterward the priest shall go in to see the house; and he shall look on the plague, and behold, if the plague be in the walls of the house with hollow strakes, greenish or reddish, and the appearance thereof be lower than the wall; then the priest shall go out of the house to the door of the house, and shut up the house seven days; and the priest shall come again the seventh day and shall look; and, behold, if the plague be spread in the walls of the house, then the priest shall command that they take out the stones in which the plague is, and cast them into an unclean place without the city; and he shall cause the house to be scraped around about, and they shall pour out the mortar that they scrape off without the city in an unclean place, and they shall take other stones and put them in the place of those stones, and he shall take other mortar and plaster the house. And if the plague come again and break out in the house after that he hath taken out the stones, and after he hath scraped the house, and after it is plastered; then the priest shall come in and look, and behold, if the plague be spread in the house, it is a fretting leprosy in the house; it is unclean. And he shall break down the house, the stones of it, and the timber thereof, and all the mortar of the house, and he shall carry them forth out of the city into an unclean place."*

The astringent, antiseptic properties of the wine of the country was well known, and the usual treatment of wounds seems to have been with wine and olive oil. Balsams from cedar and other resinous trees were largely used in the form of plasters, but the Scriptures give us few hints as to the method of treating internal maladies. The mineral springs with which Palestine abounds were made use of in many kinds of sickness, and many of them possessed virtues that seem almost miraculous, but the attention of physicians was chiefly given to injuries and skin diseases.

With the conquest of the world by Rome, and the consequent merging of all nations into one great commonwealth, the national lines of all sciences, our own included, were broken down and a condition prevailed much like the present. The Romans were never a scientific people, save in the arts of war, and those tributary to military matters, and Greek physicians were transported to the Imperial City to supply the metropolitan needs. Of these the most prominent was Galen, who was born in Pergamus, in Asia Minor, in the year 131 A. D., during the reign of the Emperor Hadrian. He received a liberal education, and so great was his native ability that at the age of eighteen, when he began his medical studies, he was already familiar with the teachings of the Stoic, Academic, Peripatetic, and Epicurean schools of philosophy. He received instruction from the most noted men of his day, and traveled into foreign countries to see the practice of other nations. Among the places visited was Alexandria, where for the first time he saw a human skeleton. His learning was famous throughout the Roman

*Leviticus,14:35, *et seq.* (Revised Verion).

empire, and the emperors, Marcus Aurelius and Lucius Verus, wished to have him with them, but he preferred traveling. His fame chiefly rests on his skill as an anatomist and as a writer. He is said to have composed nearly five hundred treatises on different subjects, including logic, ethics and grammar, besides professional topics. In the intellectual barrenness of the world at his time, his authority was so high as to overshadow all else, and in the sixteenth century, when Vesalius showed by actual dissection that some of the statements of Galen were erroneous, he brought on his head the wrath and condemnation of his cotemporaries, who claimed that the evidence of the senses was not equal in authority to the works of Galen. To him, however, we are indebted for the first appeal to nature as an infallible guide, though the philosophical tendencies of his age opposed experiment and actual demonstration. He made some physiological experiments, from which he decided that the brain was the seat of the intellectual faculties, and that its function was not merely to cool the blood as had been previously taught. He retained the erroneous doctrine of the "animal spirit," which he conceived to flow through the nerves and to be the active agent of the soul. Although many of his views were erroneous, he displayed originality and a willingness to learn that was rare among the medical men of his time and indeed for centuries afterwards.

With the fall of the imperial power of Rome came also the downfall of such learning as had been accumulated in the past. The "Dark Ages" came on, bringing with them much that is picturesque, much that is admirable, but withal a mental stillness that resembles death. The religion of the people lost the rational and logical clearness that had marked the early days of Christianity, and a superstitious veneration of holy things and holy places took its place. Relics of saints were esteemed of great efficiency in curing diseases among those who were faithful to the teachings of the Church, and outside its pale witchcraft and demonology exerted wide influence. The few earnest students that still existed were generally under the ban of the Church, and all of the sciences were so entangled with astrology and the "black arts" that little or no advance was made. Instead of chemistry, alchemy was pursued, and the talents that might have accomplished some meritorious work were frittered away on the philosopher's stone and perpetual motion. In this general obscurity but one name stands out distinctly above all others, and that one was considered for ages as the very type of a charlatan, and even now it is difficult to properly classify him. Paracelsus was born in Switzerland in 1493, and owing to his own reticence as to his early life little is known of his youth. As a chemist he was far in advance of his age. He was acquainted with hydrogen gas and with hydrochloric and sulphurous acids in the gaseous state. He distinguished alum from the "vitriols," asserting that it contained an earth, whereas the others contained metals. He described the chemical process of combustion and showed that the same reactions occurred in respiration, thus being the first student of physiological chemistry. He extracted the active principles from medicinal plants, and used them instead of the crude drug, and conferred a lasting benefit on our race by teaching that the function of chemistry is not the artificial production of gold, but the application of scientific principles to pharmacy and the industrial arts. He appealed constantly to nature as the final judge, and scouted the close adherence to Galen that prevailed at his time. He asserted that a hair of his head knew more than all the writers of his day, and that the clasps of his shoes were more learned than Galen. He traveled all over Europe on foot, talking with old women, monks, friars, Jews, Saracens, all who pretended to any knowledge of the healing arts, and undoubtedly accomplished many cures. He attacked the humoral theory of disease, and, it is claimed, foreshadowed the circulation of the blood, and many later discoveries; but it is doubtful if this is so. His wild attacks on all that was held as true at the time in their vague expressions and aimless epigrams, seem to have hidden meanings at times, but that the author knew what he meant can not be seriously held by anyone who reads the fragments of his writings that survive. He burdened himself with a mass of charlatanry. He vaunted a panacea which he styled "Azoth," "the tincture of life," and claimed the power of making man immortal, but died at the early age of forty-eight. From some source he obtained a glimpse of physiological chemistry, and asserted that all diseases were the result of chemical processes. He was a mass of contradictions, a scientist of high merit in some ways and the veriest quack in others. To him we owe the first ca l to return to nature as our guide, to study her ways and be wise, and yet he claimed the power of upsetting those laws of nature with which the most superficial study must have made him familiar. It is impossible to judge him correctly, or to measure such a mind by the standards applied to others.

In the meantime, a power was arising of which the mediæval world knew but little. In the desert lands of Arabia dwelt a race of men who for untold centuries had lived a free and lawless life, whose hands were against all men and all men's hands were against them. Holding a distorted form of the Jewish faith, the civilization and power of Hebrew, Phœnician, Greek and Roman had passed over them unchanged, but a prophet arose among them who was destined to affect the history of the world. Mohammed first converted his own people, and putting himself at the head of his army of converts began to spread the religion of Islam by sword and fire. The dying monarchies of Egypt and northern Africa offered a tempting prey, and fell readily into the hands of the invaders, and in time the whole southern shore of the Mediterranean was under the Crescent. A narrow strait separated them from Europe, and aided by internal dissensions in the country itself Musa and Tarik rapidly subdued Spain.

Some unknown influence now prevailed over the hitherto rapacious and intolerant Moors. Religious toleration was freely extended, even to the Jews who had been persecuted throughout the rest of Europe, and among the Moors themselves the arts of peace and civilization were cultivated. The Moslem power extended in an unbroken stretch from the Pyrennees to the Bosphorus, and cordial relations were established with the Greek empire, whose capital was in Constantinople. The classic writers were studied by them at a time when the rest of Europe had almost forgotten their existence, and as a result the fame of the Arabic physicians spread throughout the world. During the Crusades the western nations looked with wonder on the skill of the "barbarians" in surgery and medicine, and promptly ascribed it to the devil. For this reason the names of very few have come down to us, and I can but select one as an illustration. Avenzoar was born at the latter part of the eleventh century, in Seville, and was carefully taught the Greek and Latin languages, and through them the system of Greek philosophy. He was an indefatigable student of Galen and Hippocrates, whom he greatly admired, but criticised severely for the errors made by them in their anatomical and physiological writings. He made many experiments of which he left carefully written reports, and advocated feeding by a tube in cases in which there was difficulty in swallowing, as also nutrition by inunction. He was denounced by the authorities of the Church, and although his works were translated into Hebrew and thence into Latin, they are now very scarce and almost inaccessible.

With the exception of Constantinople, almost the only knowledge of antiquity was lodged among these Saracens. The Ottoman empire now reached almost to the walls of the Greek capital, and among the Christians fear and hatred of the Turks became so great that the former kindly intercourse was impossible. At last came the fall of the city, and the Greek scholars, with their precious manuscripts, were scattered throughout the world.

The effect of this emigration was the so-called Renaissance, a new birth of science. The well-nigh forgotten works of the ancients were greedily studied from the Danube to the Thames. The elaborate artificial system of the schoolmen fell at a blow, and art, architecture, religion, government, all underwent a revision. The discoveries of Columbus showed at once the ignorance of the old world and prepared men's minds for the changes about to come. Galileo and Copernicus gave a fatal blow to astrology, and dissection, aided by pathological research, overthrew many deep-rooted but erroneous ideas in our line of study.

Unfortunately, ours was the last science to feel the reviving breath of the Renaissance, and at the very beginning commenced that line of martyrs that constitutes our pride. Space does not permit mention of but one, but he is the type of many. Andrew Vesalius was the son of a merchant of Brussels who at the age of 14 began the study of anatomy under Duboise, a noted teacher of that day. This teacher is now remembered from three circumstances, his laziness, his avarice, and his having been the first teacher of Vesalius. According to the writings of his pupil, a human subject was never seen on his tables, and but few of the lower animals, and he slavishly followed Galen in all of the errors of that author. Vesalius first showed that the pores of Galen between the sides of the heart did not exist, and that the only communication between the two sides was through the lungs. For this he was so derided, and so many obstacles put in his way, that he went to Italy, and in 1536 he was in Venice, working with such zeal that at the age of twenty-one he was offered a professorship and requested to demonstrate publicly in Padua. For seven years he remained here, and became professor of anatomy in Bologna and Pisa, as well as Padua, delivering lectures in each university every winter. At twenty-eight, he produced his work on anatomy showing the many errors of Aristotle, Galen and Hippocrates, and then was made court physician to Charles V. Even royal power could not protect him from the persecutions of the followers of Galen. Every charge that envy could devise was brought against him, and at last "the massive vengeance of the Church" was brought on him for some offence, precisely what can not be made out. The charge was dissecting a body not yet dead, but the evidence was so contradictory that it is apparent that something else was beneath it. He was sentenced to perform a pilgrimage to the Holy Land, and sailed with a Venetian fleet, but while at Jerusalem he was invited to accept the professorship at Padua which he had surrendered to become the court physician. On his way thither he was wrecked on the island of Zante, where he died in such poverty that nothing but the charity of a goldsmith saved his body from being thrown to wild beasts.

Such then was the fate of one whom at present the world of science delights to honor. Despised, persecuted and poor, he is buried by charity, because he preferred truth to triumphant error, and chose to bear ridicule and hatred, rather than profess and teach what he knew to be wrong, although held by the masses about him to be right. Men of this type are rare, but the world can progress but little without them.

Vesalius was not the only great light that Brussels was destined to produce at this time. The sturdy race of men that the Low Countries had developed were not confined to men of war, but in the dawn of intellectual freedom some of the most heroic champions of truth arose from the plains of northwestern Europe. One to whom pathology is especially indebted is Jean Baptiste Van Helmont. He was of a noble family of Brussels, where he was born in 1577, the heir of vast possessions. At Louvrain he pursued the usual course of scholastic philosophy taught by the Jesuits in that city, but finding little to attract him in the sterile course laid down for him, he began to look beyond the curriculum for intellectual food. By one of those chances that seem predestined, he read á Kempis, and the mysticism of that author satisfied the craving of his ardent and imaginative mind. From him he learned that wisdom was a gift of the Supreme being, and was to be obtained by mortification and prayer, and from that time his life underwent a total change. Stripping himself of his wealth, he led a life of humility and study. With a view to aiding those in need, he studied medicine, mastering Hippocrates and Galen so rapidly as to astound his fellow-students. As he believed that Christianity was not merely to be believed but practiced, and to be judged by its results, so he believed that the statements of these masters of antiquity were to be tried by the same test. An opportunity soon presented itself. In some manner he contracted the itch, and turned to Galen for its cure. Galen attributes this affection to overheated bile and sour phlegm, and recommends vigorous purging as a remedy, and this Van Helmont tried most thoroughly; but as the itch got no better, he decided that Galen, wrong in one, was wrong in all, and if he was not to be trusted to whom in the medical world was he to turn? He therefore determined to carve out a system of his own, and the result was what might be expected from mystical dreamer. He asserted that in man was a principle which he called Archæus, a creative spirit, which working on water as a raw material, by means of a ferment produced all the changes that go to the growth and nourishment of the body. He claimed that digestion was not a mechanical process, nor was it caused by heat, for it is arrested in fevers and goes on in fishes and other cold-blooded animals. He claimed that Archæus commands and an acid is generated in the stomach by which the food is dissolved. The next step is the neutralization of this acid by the bile from the gall bladder, the next takes place in the vessels of the mesentery, from which the dissolved food goes to the heart and is acted on by the vital spirits, by which it is turned into material blood and goes to the brain. From thence it is transformed into the various tissues by each taking from the current what t needs, Archæus presiding over and directing the work. If in this we substitute "nutrition" for "digestion," we can not but be struck with the wonderful insight he had into the processes of nature. He claimed that all diseases were caused by perturbations of Archæus or derangements of the blood, and that many diseases, as gout for an instance, were not confined to the part in which they appeared, but were general. In this system there was no room for Galen and his theories, and he was bitterly assailed on all sides. He next turned on the alchemists, and science is indebted to him for exploding many of their pretensions. He, like Paracelsus, was a strange medley of stern practical sense and mystical philosophy. Crying bitterly for proofs of all that was claimed as true in his day, he conceived some of the wildest ideas that were ever advanced in science, but none the less he aided in teaching men to think.

By this time the belief in the infallibillity of Galen was beginning to totter on its base, and the final blow was about to fall. In 1578 William Harvey was born in the county of Kent, England, in a house that was in later times the post-house of the town of Folkestone, and which still belongs to Caius College, Cambridge, to which Harvey bequeathed it. His father was a yeoman, but sufficiently prosperous to be able to send his son to the Canterbury grammar school. From there he entered Caius College as a pensioner, and at the age of nineteen received the B. A. degree. Five years later he was made a doctor of medicine at Padua, and returned to his native land, where thirteen years later he became Lumleian lecturer in St. Bartholomew's Hospital. In 1616 he began his lectures on the circulation, and for the first time asserted that the blood made a regular circuit through the body, passing from the heart through the arteries, and returning through the veins. To this conclusion he was led by the arrangement of the cardiac veins, the fact that the pulse and heart beat were synchronous, the arrangement of valves in the veins permitting flow in but one direction, the observed fact that if a band were tied about a limb the veins would fill from below, and that if a vein were severed the blood flowed in a steady stream, but if an artery were cut the flow was in jets corresponding to the throbs of the heart. As a piece of logical reasoning this classic treatise of some sixty-seven pages is unsurpassed in the world, and in every point assertion was sustained by demonstration except one. He was not able to demonstrate the capillaries, but so forcible was his reasoning that when, in 1661, Malpighi, with the newly invented microscope, saw the blood passing from one set of vessels to the other, it but confirmed what had by that time become the accepted doctrine.

It is difficult to imagine the furore created by the enunciation of this doctrine. The shades of all the fathers were invoked against him, personal and professional attacks of all sorts were made on him, and his practice fell to almost nothing. In France he was styled a circulatoir, a name applied to the wandering (circulating) quacks that frequented country fairs and markets, but few of his adversaries showed the wit or good humor indicated by this title. In the attacks made on him abroad he was fortunate, for the English physicians rallied to his support, and by witnessing his experiments became supporters of the truth of his deductions. The confirmation of his great discovery was made, as I have said above, by Malpighi, with whose name you will become very familiar before the session is over. This man, the next in our roll of honor, was the first to apply the microscope to the medical sciences, and so many and so great were his discoveries that it is not surprising that they were greeted with incredulity by his cotemporaries. He was born about twenty miles north of Bologna, in 1628, and was educated in the University of that city in all the classical scholarship of the day. In 1649 his family became impoverished, and it was necessary for him to choose a profession, and, by a chance to which the world is indebted, he selected medicine. Then, as now, the liberal education received in his early years proved of signal advantage, and so great was his proficiency that in 1656 he was appointed to the chair of theoretical medicine in Pisa. Ill health, induced by hard study, compelled him to leave Pisa after four years, but he was induced to accept a position in Messina for four years at a salary of one thousand scudi (about $960 in our money), at the expiration of which term he returned to his native University, where he labored for twenty-five years. In 1691 his health, always delicate, gave way altogether, and Pope Innocent XII appointed him his private physician, by way of giving him an honorable sinecure in retirement, and death befell him three years later in the Vatican.

Malpighi was among the first to practice vivisection, and it occurred to him to use the compound microscope for carrying on his investigations. So faulty was the construction of these instruments that a reaction took place against them, and the simple lens was chiefly used. The perfection to which some of these lenses were brought is very remarkable, a collection of which that until recently was in the possession of the Royal Society of London, being equal to any made to-day. In examining the lung of a frog in 1661, four years after Harvey's death, to his delight he saw the blood corpuscles passing from artery to vein, completing the pulmonary circulation. This was hailed only as a confirmation of an idea then generally accepted, but his next discovery was a genuine advance. The conception of glandular tissue that had prevailed was that a gland was essentially a coil of blood-vessels, out of which the secretion exuded, but Malpighi showed that the type was that of a tube lined with a membrane that elaborated the peculiar product of the gland. His name is associated with the lower layer of the skin, the vascular coils of the kidney, and the follicular bodies of the spleen, all of which he demonstrated, and but little more has been done for them since. His researches into the histology of the brain led him into a serious error, for he thought the brain to be a vast gland that secreted the "vital spirit," and made many lamentable mistakes by trying to twist facts to fit his theory instead of adapting his theory to suit the facts. In one who accomplished so much, latitude should be given for some errors, and it is almost incomprehensible how he could have done so much accurate work without the staining agents that are now essential, and yet how, having done so much, he did not anticipate Schwann in discovering the properties of cells.

More than once in the history of the development of our science have great advances been made by those without the limits of our profession, even after it became a compactly organized body. One of the first and greatest of these was

Leuwenhoek. Born at Delft, in Holland, in 1632, he was brought up to the trade of a glass grinder, in the pursuit of which calling he learned to make and use lenses. He was one of those rare geniuses whose native ability seemed able to overcome all disadvantages occasioned by the lack of preliminary training, and at the age of twenty-eight published an account of the capillary circulation, as he had observed it with his microscope in the tail of a young eel. Eight years later, when the Czar Peter the Great passed through Delft, he requested Leuwenhoek to show him this phenomenon, by which, says the old chronicle, "His Majesty was greatly pleased and amazed."

Besides completing the chain of evidence by which Harvey's great discovery was established, he contributed more than any other naturalist to the overthrow of the doctrine of spontaneous generation and enunciated the saying, that is now a dogma of science: "*Omne vivum, ex ovo*," all life not merely from antecedent life, but from the same kind of life. This he established by careful and prolonged observation in a great variety of cases in which "spontaneous generation" was said to have occurred, especially in the lowest forms of life, which were said to have been "bred from corruption." He traced the full history of oak-galls, and showed that they are the result of the insertion of an egg by a special kind of an insect into the tree, thus exciting a peculiar vegetable growth. He showed that weevils are hatched in and not from grain, and are only grubs hatched from eggs deposited by winged insects. His most celebrated investigation was into the reproduction of eels, which at that time were supposed, even by learned men, to be produced from dew, a notion that survives in the popular childish belief that horse-hairs turn to snakes when immersed in water. He died at the age of ninety-one, after being elected a member of several of the most prominent learned societies of the time, among them the Royal Society of England and the Academy of Sciences of Paris.

This, gentlemen, brings our science down almost to our own time, for with the introduction of the microscope begins the era of modern medicine. The discoveries made since then will be discussed as we come to them in the course of our studies during the term, and with this introduction I hope you will be better able to appreciate the work of to-day.

